# Secondary oxalosis induced by xylitol concurrent with lithium-induced nephrogenic diabetes insipidus: a case report

**DOI:** 10.1186/s12882-020-01814-9

**Published:** 2020-05-01

**Authors:** Shinobu Takayasu, Aya Kamba, Kazutaka Yoshida, Ken Terui, Yutaka Watanuki, Noriko Ishigame, Satoru Mizushiri, Tetsu Tomita, Kazuhiko Nakamura, Norio Yasui-Furukori, Makoto Daimon

**Affiliations:** 1 Department of Endocrinology and Metabolism, Hirosaki University Graduate School of Medicine and Hospital, 5 Zaifu-cho, Hirosaki, Aomori, 036-8562 Japan; 2 Department of Neuropsychiatry, Hirosaki University Graduate School of Medicine and Hospital, 5 Zaifu-cho, Hirosaki, Aomori, 036-8562 Japan; 3 Department of Neuropsychiatry, Dokkyo Medical University School of Medicine, 880 Kitakobayashi, Mibu, Shimotsuga, Tochigi, 321-0293 Japan

**Keywords:** Oxalosis, Xylitol, Nephrogenic diabetes insipidus, Lithium

## Abstract

**Background:**

Xylitol is an approved food additive that is widely used as a sweetener in many manufactured products. It is also used in pharmaceuticals. Secondary oxalosis resulting from high dietary oxalate has been reported. However, reported cases of oxalosis following xylitol infusion are rare.

**Case presentation:**

A 39-year-old man with a 16-year history of organic psychiatric disorder was hospitalized for a laparoscopic cholecystectomy because of cholecystolithiasis. He had been treated with several antipsychotics and mood stabilizers, including lithium. The patient had polyuria (> 4000 mL/day) and his serum sodium levels ranged from 150 to 160 mmol/L. Urine osmolality was 141 mOsm/L, while serum arginine vasopressin level was 6.4 pg/mL. The patient was diagnosed with nephrogenic diabetes insipidus (NDI), and lithium was gradually discontinued. Postoperative urine volumes increased further to a maximum of 10,000 mL/day, and up to 10,000 mL/day of 5% xylitol was administered. The patient’s consciousness level declined and serum creatinine increased to 4.74 mg/dL. This was followed by coma and metabolic acidosis. After continuous venous hemodiafiltration, serum sodium improved to the upper 140 mmol/L range and serum creatinine decreased to 1.25 mg/dL at discharge. However, polyuria and polydipsia of approximately 4000 mL/day persisted. Renal biopsy showed oxalate crystals and decreased expression of aquaporin-2 (AQP2) in the renal tubules. Urinary AQP2 was undetected. The patient was discharged on day 82 after admission.

**Conclusions:**

Our patient was diagnosed with lithium-induced NDI and secondary oxalosis induced by excess xylitol infusion. NDI became apparent perioperatively because of fasting, and an overdose of xylitol infusion led to cerebrorenal oxalosis. Our patient received a maximum xylitol dose of 500 g/day and a total dose of 2925 g. Patients receiving lithium therapy must be closely monitored during the perioperative period, and rehydration therapy using xylitol infusion should be avoided in such cases.

## Background

Xylitol is an approved food additive used as a sweetener in many products, such as dietary supplements, toothpaste, and chewing gum. It is also widely used in pharmaceuticals. No health risks associated with normal levels of xylitol consumption have been reported, and no limit in its daily intake has been specified [1]. Use of products containing xylitol promotes improved dental health, digestive function, body weight, and diabetes management [2, 3]. In Japan, intravenous xylitol infusion is sometimes used to maintain fluid balance and caloric intake when enteral intake is insufficient, especially in patients with diabetes mellitus. Xylitol is also used as a diluent for compatible medicinal products for parenteral administration in this country. We are unaware of any potential adverse effects.

Secondary oxalosis results from increased dietary oxalate intake, increased intestinal oxalate availability, or decreased intestinal oxalate degradation. Excessive intake of foods containing oxalate precursors, which are found in some vegetables, fruits, tea, and vitamin C, is a reported cause of secondary oxalate nephropathy [4, 5]. However, there are few reported cases of oxalosis following xylitol infusion [6–8].

Lithium is widely used and is associated with increased risk of tubular dysfunction, which leads to urine concentration defects and subsequent nephrogenic diabetes insipidus (NDI). NDI occurs in approximately 20 to 40% of patients receiving chronic lithium therapy [9].

Herein, we describe a case of lithium-associated NDI with secondary oxalosis induced by excess xylitol infusion. NDI was undiagnosed in the patient before admission because polydipsia had compensated for polyuria. Decreased renal concentrating capacity became apparent perioperatively because of fasting, and renal and presumably cerebral oxalosis developed because of excess xylitol infusion. This case highlights the potential risks of xylitol infusion in patients who require high-volume infusions.

## Case presentation

A 39-year-old man presented with abdominal pain and jaundice and was diagnosed with cholecystolithiasis. He had a 16-year history of organic psychiatric disorder induced by a large hematoma and contusion of the brain following injury in a traffic accident. Because of delusions of persecution and severe irritability, the patient was treated with several antipsychotics and mood stabilizers, including zotepine (400 mg/day for 6 years), risperidone (12 mg/day for 8 years), paliperidone (12 mg/day for 3 years), olanzapine (20 mg/day for 8 years), and lithium (1200 mg/day for 6 years). His serum lithium concentration ranged from 0.75 to 1.35 mEq/mL.

**Fig. 1:**
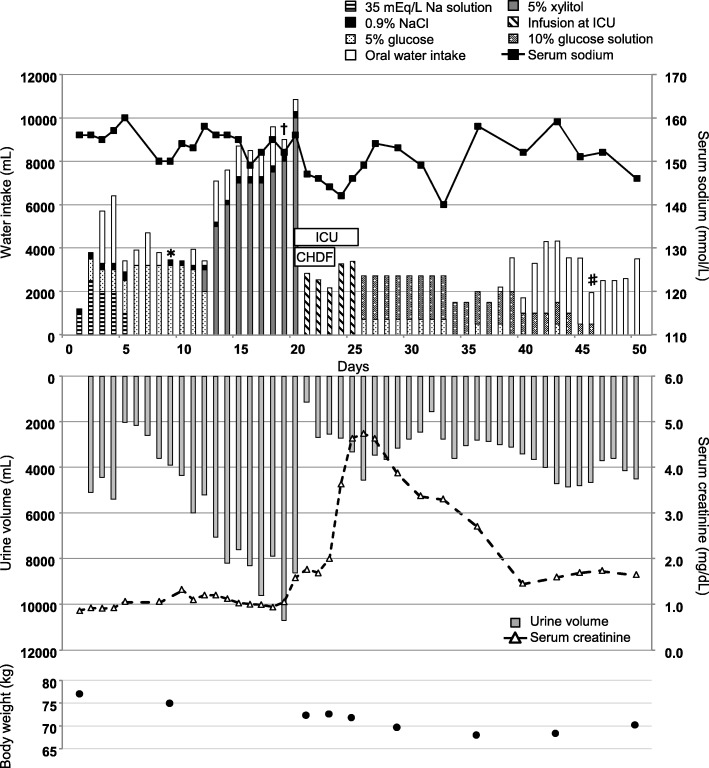
Clinical course of the patient. Graphs present changes in clinical and laboratory findings over time. ICU, intensive care unit; CHDF, continuous hemodiafiltration. * surgery; † discontinuation of lithium; ♯ discontinuation of intravenous infusion

The patient’s clinical course is shown in Fig. 1. At admission, the patient’s height was 176.1 cm and his body weight was 77.1 kg. His blood pressure was 136/102 mmHg and his pulse rate was 111 beats per minute. His body temperature was 37.6 °C on admission (Day 1). Laboratory examinations on Day 2 revealed a white blood cell count of 11,480/mL (range 3500–8500), C-reactive protein of 2.89 mg/dL (range 0.00–0.30), aspartate transaminase of 130 U/L (range 13–33), alanine transaminase of 245 U/L (range 8–42), alkaline phosphatase of 692 U/L (range 115–359), lactate dehydrogenase of 230 U/L (range 119–229), total bilirubin of 6.3 mg/dL (range 0.3–1.2), γ-glutamyltransferase of 348 U/L (range 10–47), amylase of 128 U/L (range 37–125), serum creatinine of 0.92 mg/dL (range 0.6–1.1), urea nitrogen of 5 mg/dL (range 8–22), serum sodium of 156 mmol/L (range 138–146), serum potassium of 3.7 mmol/L (range 3.6–4.9), and serum chloride of 114 mmol/L (range 99–109).

Because the patient’s polyuria was > 4000 mL/day, he received 3200 mL/day of 5% glucose perioperatively (Days 6–12). However, his serum sodium levels remained between 150 and 160 mmol/L. Plasma and urine osmolality were 318 mOsm/L and 141 mOsm/L, respectively, while the serum arginine vasopressin level was 6.4 pg/mL (range 0.0–3.8). Desmopressin was ineffective, resulting in a diagnosis of NDI. Nine days after admission (Day 9), laparoscopic cholecystectomy was performed. We gradually withdrew lithium starting on Day 15 because it was the suspected cause of NDI. Lithium was completely discontinued on Day 19. Urine volume increased further to a maximum of 10,000 mL/day. Because the patient had free water excretion resulting from NDI and we wanted to avoid excessive glucose to prevent diabetes mellitus, rehydration therapy with xylitol infusion was initiated. A maximum of 10,000 mL/day of 5% xylitol was administered for 8 days (Days 13–20).

The patient’s serum lithium concentration was 0.89 mEq/mL on Day 15. He developed lethargy on Day 19, although laboratory examinations showed no remarkable change (serum creatinine = 1.05 mg/dL, urea nitrogen = 8 mg/dL, serum sodium = 152 mmol/L). His consciousness level declined and his Glasgow Coma Scale (GCS) score was 6 points (E4V1M1) with metabolic acidosis on Day 20. The patient did not show meningeal signs, involuntary movements, or generalized tonic–clonic seizures. He had decreased muscle tension/tonus and deep tendon reflexes but showed jaw jerk reflex and snout reflex; a brainstem lesion was suspected. The patient maintained spontaneous breathing.

Arterial blood gas testing without oxygen therapy on Day 20 showed the following: fraction of inspired oxygen 0.21 (room air), pH 7.28 (range 7.36–7.44), carbon dioxide partial pressure = 14.7 (range 35.0–45.0), partial pressure of oxygen in arterial blood = 117.0 (range 75.0–95.0), hydrogen carbonate ion = 6.7 (range 22.0–28.0), and base excess = − 18.7 (range 23.0–27.0). Laboratory examinations revealed the following: serum creatinine = 1.58 mg/dL, urea nitrogen = 9 mg/dL, serum sodium = 155 mmol/L, serum potassium = 3.5 mmol/L, and serum chloride = 125 mmol/L. Computed tomographic and magnetic resonance imaging on Day 20 revealed brain edema but no cerebral herniation or findings of osmotic demyelination syndrome. Continuous hemodiafiltration (CHDF) was temporarily performed from Days 20 to 23, which corrected the patient’s metabolic acidosis. His consciousness level improved and his GCS rose to 12 points (E4V3M5) by Day 23. At that time, the patient showed normal brain stem reactions and deep tendon reflexes. He responded to a handshake. His serum lithium concentration was 0.05 mEq/mL on Day 22. However, his urine volume remained at approximately 3000 mL/day, serum sodium remained in the 150 mmol/L range, and serum creatinine increased to 4.74 mg/dL on Day 26. Urinalysis showed no remarkable findings throughout the examination period.

**Fig. 2:**
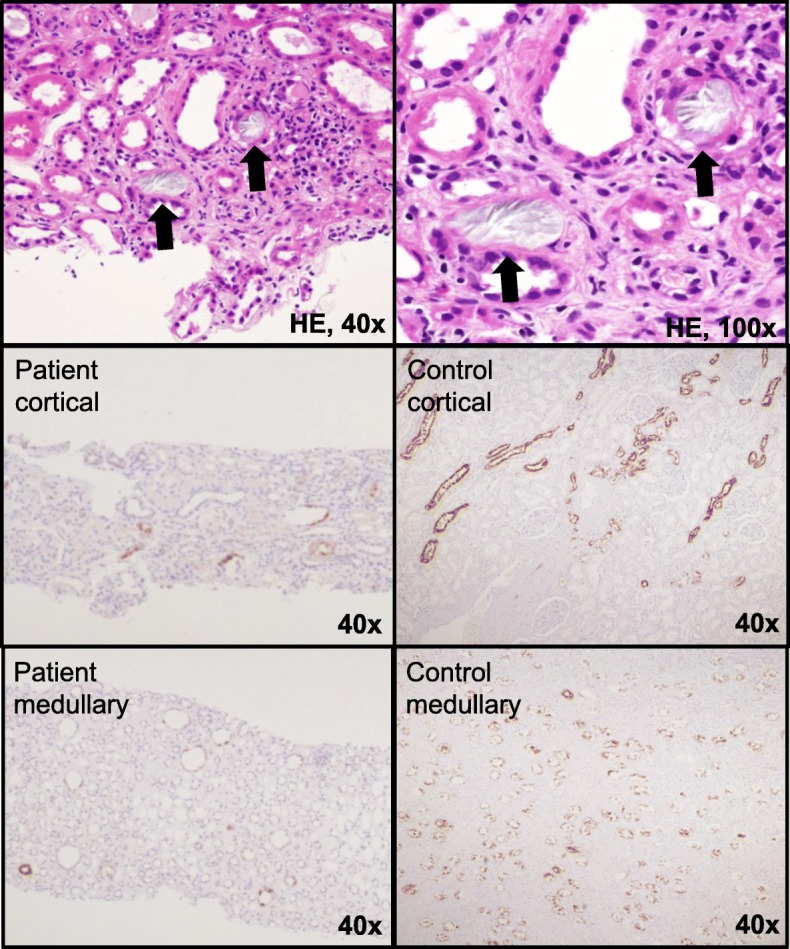
Polarized light microscopy showing double-refractive crystals deposited in cortical renal tubules. The crystals have a rosette-like structure (upper panel, arrows). Decreased levels of aquaporin-2 expression are observed in cortical (middle-left panel) and medullary (lower-left panel) renal tubules compared with control (middle and lower-right panel)

The patient’s serum sodium was in the high 140 mmol/L range and his creatinine level was near 1.5 mg/dL on Day 50. However, polyuria and polydipsia of approximately 4000 mL/day persisted. Renal biopsy on Day 60 showed deposition of double-refractive crystals in the cortical renal tubules. Tubular atrophy, macrophage and lymphocyte infiltration, and interstitial fibrosis were observed in parts of the tubulointerstitium. Decreased expression of water channel aquaporin-2 (AQP2) was observed in the cortical and medullary renal tubules (Fig. 2); no urinary AQP2 was detected on ELISA (Human Aquaporin 2 ELISA Kit, Otsuka Pharmaceutical Co., Ltd., Tokyo, Japan). The diagnosis was lithium-induced NDI and cerebrorenal oxalosis induced by excess xylitol infusion.

Laboratory examinations on Day 80 revealed the following: serum creatinine = 1.25 mg/dL, urea nitrogen = 7 mg/dL, serum sodium = 147 mmol/L, plasma osmolality = 297 mOsm/L, and urine osmolality = 106 mOsm/L. The patient was discharged 82 days after admission; his thirst gradually decreased to 1500 to 2000 mL of water per day by 2 months after discharge. Urinary AQP2 levels increased to 1.14 ng/mg/g of creatinine and serum creatinine decreased to a low of 0.90 mg/dL. However, plasma and urine osmolality were 292 mOsm/L and 62 mOsm/L, respectively, 4 months after discontinuing lithium. The patient has had serum creatinine levels of approximately 1.2 mg/dL since that time.

## Discussion and conclusions

Lithium in bipolar disorder serves as a mood stabilizer. It is also used as an antidepressant medication. Long-term lithium therapy results in decreased renal concentrating capacity, which may result in NDI [10]. Decreased urinary concentrating capacity is not rare: NDI occurs in up to 40% of patients receiving lithium [9], particularly in those undergoing long-term therapy. Long-term therapy with lithium may be associated with tubulointerstitial nephritis [11], which progresses slowly. Several reports have described occult lithium-induced NDI that was revealed during the perioperative period [12–17]. Decreased renal tubular function may be underdiagnosed prior to surgery because normal water homeostasis in patients with NDI is maintained by polydipsia. Fasting during the perioperative period may reveal decreased renal concentrating capacity, and patients may develop dehydration and hypernatremia.

Several potential mechanisms may be involved in lithium-induced NDI [18]. Importantly, however, the mechanism likely involves the inhibition of AQP2 phosphorylation. When AQP2 is phosphorylated, it translocates from intracellular vesicles of renal tubular cells to the apical membrane of the principal cells of the collecting duct, leading to reabsorption of water. Lithium induces AQP2 inactivation and decreased AQP2 expression, resulting in inhibition of water reabsorption at the collecting duct [18]. Renal biopsies in our patient revealed decreased expression of AQP2 in renal tubules and AQP2 was not detected in his urine. Both current and past use of lithium have been associated with NDI. NDI may persist despite cessation of treatment, suggesting irreversible renal damage (9, 15). In our patient, urinary AQP2 levels became detectable 4 months after discontinuing lithium. However, his serum sodium level remained in the upper 140 mmol/L range and his urine osmolality was approximately 100 mOsm/L. It remains to be determined whether decreased AQP2 and NDI are fully reversible after lithium discontinuation.

Primary oxalosis is an autosomal recessive disease. It is caused by a defect in glyoxylate metabolism because of a deficiency in the liver peroxisomal enzyme alanine glyoxylate aminotransferase, as in type 1 primary hyperoxaluria. This deficiency results in increased urinary excretion of oxalate, which forms insoluble calcium salts that accumulate in the kidneys and other organs. Recurrent urolithiasis and nephrocalcinosis with progressive loss of renal function are the main symptoms. One-half of patients exhibit initial symptoms by the age of 5 years [19]. Primary oxalosis was excluded in our patient because he had normal renal function before surgery and showed acute kidney injury (AKI) 11 days after surgery. Additionally, computed tomography showed no urolithiasis or nephrocalcinosis.

Secondary oxalosis results from increased dietary oxalate intake, increased intestinal oxalate availability, or decreased intestinal oxalate degradation. Excessive oxalate accumulation induced by exogenous factors, such as excessive vitamin C administration, ethylene glycol intoxication, the use of methoxyflurane, and infusions containing sugar surrogates [4, 6, 20], have been reported.

Patients with oxalosis sometimes develop acute renal failure and coma. A few cases of fatal oxalosis following xylitol infusion were reported during the 1970s and 1980s [7, 8]; patient autopsies showed calcium oxalate crystals in renal and cerebral vessel walls, a condition known as “cerebrorenal oxalosis.” Lumlertgul et al. performed a systematic review of case reports and case series of secondary oxalate nephropathy and reported that no patients achieved complete kidney recovery, although 41% achieved partial kidney recovery. In total, 55% of patients with oxalate nephropathy required dialysis and 49% had dialysis-dependent kidney failure. The mortality rate in that review was 51%. Urinary oxalate crystals were identified in only 26% of patients with secondary oxalate nephropathy. However, all patients had oxalate crystal deposition in the tubules and/or interstitium upon kidney biopsy. Other kidney biopsy findings included acute tubular injury, tubular damage and atrophy, interstitial mononuclear cell infiltration, and glomerular changes [5].

We did not use contrast agent after the operation. Therefore, a diagnosis of contrast-induced nephropathy was excluded. A diagnosis of acute tubular necrosis after arterial hypotension was also excluded because the patient’s circulatory and respiratory dynamics were preserved intraoperatively. The patient had shown chronic hypernatremia and no remarkable change in serum sodium or creatinine level until Day 20 (11 days after surgery), at which point he was diagnosed with AKI with metabolic acidosis. Therefore, we considered accumulation of oxalate crystal deposition in the renal vessel walls, tubules, and/or interstitium to be a possible contributing factor in AKI.

Our patient’s consciousness level declined, followed by coma. He had brain edema before correction of hypernatremia. Hypernatremia was not corrected quickly. All patients with cerebral oxalosis have brain edema and autopsies have shown calcium oxalate crystals in the cerebral vessel walls. To the best of our knowledge, there have been no reports of non-fatal cerebral oxalosis. Because our patient had a previous history of brain contusion following injury in a traffic accident, he had more intracranial space than usual. This may have provided protection against excessive intracranial pressure. An alternative possible cause of declining consciousness in our patient is lithium intoxication. Although the patient’s serum lithium concentration was in the normal range on day 15, it might have been higher when he developed impaired consciousness. CHDF would have been an effective treatment in that situation [21].

Our patient had persistent hypernatremia with no remarkable change in serum sodium or creatinine levels until day 20, at which time he developed AKI with metabolic acidosis. Urinary oxalate crystals were not identified. Unfortunately, oxalate and its metabolites were not measured in the plasma or urine. However, there was tubular crystal deposition, tubular atrophy, and interstitial inflammation in the kidney. These findings and clinical manifestations are consistent with cerebrorenal oxalosis. Immediate initiation of CHDF can correct metabolic acidosis and water balance and may prevent further deposition of calcium oxalate in organs and vessel walls, resulting in quicker recovery of consciousness and renal function.

Xylitol is converted to xylulose 1-phosphate through the actions of o-xylulose reductase and fructokinase. Aldolase converts xylulose 1-phosphate to dihydroxyacetone phosphate and glycolaldehyde, the latter being a substrate for the two-carbon pathway [22]. McWhinney et al. showed that the 16-g oral xylitol loading test did not increase oxalate excretion significantly in either stone formers or normal subjects [23]. In contrast, Bais et al. purified ketohexokinase and aldolase from human liver and examined their properties. With D-xylulose as substrate, ketohexokinase and aldolase can catalyze a reaction sequence that forms glycolaldehyde, a known precursor of oxalate. They demonstrated that increased xylitol metabolism in human liver occurs under conditions of excessive xylitol intake, such as in parenteral infusion, and increased oxalate production may result in oxalosis [24]. Xylitol is an approved food additive used as a sweetener in many products, such as dietary supplements, toothpaste, and chewing gum. The acceptable daily intake of xylitol is not specified by the Food and Agriculture Organization of the United Nations/World Health Organization Joint Expert Committee on Food Additives. On the basis of a few cases of renal oxalosis following xylitol infusion in the 1970s and 1980s [25–27], less than 100 g/day of xylitol infusion is recommended in Japanese drug guidelines. However, the guidelines do not mention a maximum dosage under contraindications. We had not experienced adverse effects with xylitol infusion before this case. In addition, we were unaware of the recommended limit and any potential adverse effects of xylitol infusion. As a result, our patient received a maximum dose of 500 g/day and a total dose of 2925 g. The excess xylitol intake could have resulted in cerebrorenal oxalosis in our patient.

In summary, NDI was undiagnosed preoperatively in our patient because polydipsia compensated for polyuria. Decreased renal concentrating capacity became apparent perioperatively because of fasting, and an overdose of xylitol infusion then led to cerebrorenal oxalosis. We emphasize that patients receiving lithium therapy must be closely monitored perioperatively to detect un.

diagnosed NDI. Rehydration therapy using xylitol infusion should be avoided, particularly in patients with renal free water excretion, as can occur with NDI.

## Data Availability

All data generated during this report are included in this published article.

## Abbreviations

AQP2: Aquaporin-2
NDI: Nephrogenic diabetes insipidus
